# Annotations

**Published:** 1907-05-04

**Authors:** 


### Annotations.

### Notification of Births Bill.

The purpose of this Bill, which was introduced into the House of Commons by Lord Robert Cecil on April 23, is to make more efficient the existing machinery for the preservation of infant life. Under the present law, registration of the birth of a child may be effected any time within six weeks after the date of the birth. It is now proposed to reduce this interval to a period of forty-eight hours. Further, to facilitate this procedure, it is provided by the Bill that notification may be accomplished in the simplest possible manner—a mere postcard to the medical officer of health will be sufficient. Responsibility for the necessary intimation is thrown in the first place on the father of the child, and, failing him, on anyone "who has been in attendance on the mother"—a phrase, it must be noted, which may manifestly include the medical practitioner in charge of the case. It is hoped that prompt and early registration of births will give an increased opportunity to the various agencies which exist for the purpose of guiding ignorant mothers in the feeding and rearing of their children. At present the late date for registration keeps these agencies, in many instances, ignorant of the fact of a child's birth until, perhaps, irreparable mischief has been accomplished. To illustrate the truth of this statement it is sufficient to say that one-third of the children dying in the first year of life die under the age of six weeks, that is, they die before the fact of their birth has necessarily been subject to legal registration. Beyond question, much of the terrible infantile mortality of some of our large towns is due to ignorance on the part of mothers and of other women who profess to be skilled in the nursing and rearing of children. In certain districts, societies which have been formed for the purpose of dispelling this ignorance, can already show good results, and Lord Robert Cecil's Bill will, if it becomes law, considerably aid operations that are based on the claims both of humanity and national interest. There is in the Bill another provision which is of great value, namely, a clause demanding the compulsory registration of still-births.

### A Discussion on Addison's Disease.

The recent discussion at the Royal Medical and Chirurgical Society has not added very much to our knowledge of the general features of Addison's disease. Several speakers insisted on the difficulties of diagnosis due, in one group, to scanty or absent pigmentation, and in another group to considerable pigmentation with little or no development of the evidences of asthenia. These difficulties are widely recognised by experienced clinicians, and individual examples, both at one extreme and the other, were quoted in the discussion above referred to. The position seems to be, what indeed was pointed out years ago, that in the most acute cases death occurs before there is time for marked pigmentation to develop, whilst in cases of a chronic order pigmentation is the most prominent clinical event. But though the discussion consisted mainly in the presentation of well-recognised facts, it was by no means uninteresting, and it had at least one valuable feature. It illustrated the value of certain new and exact methods of observing abnormal clinical developments. Thus Dr. Otto Grünbaum showed that the estimation of the blood pressure when associated with the administration of suprarenal extract gave information regarding the functions of the suprarenal bodies. He has found that the extract administered by the mouth to normal individuals has no effect on the blood pressure. Hence when such administration is followed by a rise in blood pressue it may be fairly argued that a condition of suprarenal inadequacy is present, and there is thus a presumption in favour of Addison's disease. Such an observation is of obvious clinical value. Again, Dr. Grünbaum pointed out that by observations on the opsonic index, in connection with the inoculation of tuberculin, it may be possible to decide that a lesion of the suprarenals is of a tuberculous nature. These methods make for exactness in diagnosis, and are therefore to be welcomed.

### The Care of Patients.

In a characteristically reasonable and commonsense address recently delivered to the North London Medical and Chirurgical Society, Dr. James Grey Glover discussed, with many happy allusions, the relation of medical practice to the treatment of minor ailments and to the promotion of health and long life. These ends are in some senses less urgent and less attractive than the therapeutic problems presented by cases of acute disease. But they are among the most important of the functions of the medical practitioner, and it is pleasing to find them exalted to their true position by one who speaks from the authoritative basis of a long and honourable experience. Acute diseases, as Dr. Glover reminds us, do not form the bulk of medical work. The smaller complaints—trivial, perhaps, to the exponent of the newest pathology—constitute a large and serious handicap and embarrassment on human happiness and activity, and the earnest and serious study of them is the duty not less than the opportunity of the practitioner. It is in this direction that so much complaint is made of the invasion of medical practice by unqualified prescribers, to meet which, at least in some quarters, help is prayed from the Parliamentary Jupiter. We have often ventured to say that such unqualified practice cannot be suppressed by law, and, naturally therefore, we welcome Dr. Glover's presentation of the same conclusion. It is to the cultivation of increased efficiency on their own part, and to the influence of extending intelligence among the people generally, that the medical profession must look for an improved appreciation of the value of medical services to the body politic.

